# Investigating the Effect of Imputed Structural Variants from Whole-Genome Sequence on Genome-Wide Association and Genomic Prediction in Dairy Cattle

**DOI:** 10.3390/ani11020541

**Published:** 2021-02-19

**Authors:** Long Chen, Jennie E. Pryce, Ben J. Hayes, Hans D. Daetwyler

**Affiliations:** 1 Agriculture Victoria, AgriBio, Centre for AgriBioscience, Bundoora, VIC 3083, Australia; longchen0513@gmail.com (L.C.); jennie.pryce@agriculture.vic.gov.au (J.E.P.); b.hayes@uq.edu.au (B.J.H.); 2 School of Applied Systems Biology, La Trobe University, Bundoora, VIC 3083, Australia; 3 Queensland Alliance for Agriculture and Food Innovation, Centre for Animal Science, The University of Queensland, St. Lucia, QLD 4067, Australia

**Keywords:** genome sequence, structural variants, accuracy, genome-wide association studies, genomic prediction, genomic selection

## Abstract

**Simple Summary:**

Structural variants are large changes to the DNA sequences that differ from individual to individual. We discovered and quality-controlled a set of 24,908 structural variants and used a technique called imputation to infer them into 35,588 Holstein and Jersey cattle. We then investigated whether the structural variants affected key dairy cattle traits such as milk production, fertility and overall conformation. Structural variants explained generally less than 10 percent of the phenotypic variation in these traits. Four of the structural variants were significantly associated with dairy cattle production traits. However, the inclusion of the structural variants in the genomic prediction model did not increase genomic prediction accuracy.

**Abstract:**

Structural variations (SVs) are large DNA segments of deletions, duplications, copy number variations, inversions and translocations in a re-sequenced genome compared to a reference genome. They have been found to be associated with several complex traits in dairy cattle and could potentially help to improve genomic prediction accuracy of dairy traits. Imputation of SVs was performed in individuals genotyped with single-nucleotide polymorphism (SNP) panels without the expense of sequencing them. In this study, we generated 24,908 high-quality SVs in a total of 478 whole-genome sequenced Holstein and Jersey cattle. We imputed 4489 SVs with R2 > 0.5 into 35,568 Holstein and Jersey dairy cattle with 578,999 SNPs with two pipelines, FImpute and Eagle2.3-Minimac3. Genome-wide association studies for production, fertility and overall type with these 4489 SVs revealed four significant SVs, of which two were highly linked to significant SNP. We also estimated the variance components for SNP and SV models for these traits using genomic best linear unbiased prediction (GBLUP). Furthermore, we assessed the effect on genomic prediction accuracy of adding SVs to GBLUP models. The estimated percentage of genetic variance captured by SVs for production traits was up to 4.57% for milk yield in bulls and 3.53% for protein yield in cows. Finally, no consistent increase in genomic prediction accuracy was observed when including SVs in GBLUP.

## 1. Introduction

Whole-genome sequences have facilitated the study of large-scale genomic variations. Structural variations (SVs) are large DNA segments of deletions, duplications, copy number variations (CNVs), inversions and translocations in a re-sequenced genome compared to a reference genome [1,2]. Numerous studies have reported associations of SVs with disease traits in humans [3,4,5] and some production and disease traits in livestock [6,7,8,9,10,11,12,13]. SVs have been found to be associated with complex traits in various livestock breeds, such as nematode resistance in Angus cattle [7], fertility and milk production in Nordic Red cattle [8], and abdominal fat content in chickens [11]. As the number of individuals of a species that are whole-genome sequenced increases, the potential to reveal the spectrum of SVs for that species increases.

Genotype imputation is a strategy to infer missing genetic markers of individuals that are only genotyped on lower-density panels from a reference set of individuals genotyped on denser panels [14]. Many studies have used imputed SNP data to improve the power of genome-wide association studies (GWAS) and to increase genomic prediction accuracy [15,16,17] due to increased density of genetic markers. The process has also been applied in humans to impute SV genotypes from SNP arrays [18]. Therefore, imputation of SVs can be an alternative cost-effective solution to recover SV genotypes for large populations and thus may provide more power for downstream analysis.

Although SVs have been found to be associated with complex traits in various livestock breeds, there is limited knowledge of how much genetic variation is explained by SVs in dairy cattle for complex traits, including milk production traits and fertility. Including SVs in genomic prediction could potentially improve the accuracy of genomic estimated breeding values (GEBV) if SVs explain a proportion of the total genetic variance. In this study, we first detected high-quality SVs using two SV detection programs, Breakdancer [19] and Pindel [20], from 478 whole-genome sequenced Holstein and Jersey bulls. We then constructed five sets of SVs from 516 Holstein and Jersey animals with whole-genome sequence data and tested the accuracy of imputation and compared the performance of two imputation programs: FImpute 2.2 [21] and Eagle2.3 [22] +Minimac3 [23]. We imputed this selected set of SVs in 35,568 Holstein and Jersey cattle and performed several analyses: (1) single-trait GWAS for SVs for milk production traits, fertility and overall type and meta-analyses of GWAS results for production traits; (2) estimating the genetic variance of SVs for phenotypic traits using genomic best linear unbiased prediction (GBLUP) model; and (3) assessing the accuracy of genomic prediction in Holstein and Jersey populations using SNPs with or without imputed SV genotypes.

## 2. Materials and Methods

### 2.1. Sequencing Data

The animal samples and the whole-genome sequence data were available from the 1000 Bull Genomes Project (Run 5) [24,25]. There were 450 Holstein animals (of which 35 were sequenced twice) and 66 Jersey animals (of which 3 were sequenced twice). The sequencing reads were aligned to *Bos taurus* assembly UMD3.1 with the Burrows–Wheeler Aligner (BWA) method [26]. A large proportion of the sequences used in this study have been described and published by Daetwyler et al. (2014) [24]. Of these animals, a total of 200 animals (140 Holsteins and 60 Jerseys) were also genotyped on the BovineHD BeadChip. A total of 478 whole-genome sequenced individuals (415 Holstein and 63 Jerseys) were used as the reference for imputation. The genome coverage and insert size are summarised in Table S1. SNP genotypes of 632,003 SNPs were available for a total of 4908 bulls (3903 Holstein and 1005 Jersey) and 30,867 cows (6177 pure Jerseys, 22,903 pure Holsteins, 125 quarter Holstein, 563 half Holstein/Jersey and 1099 quarter Jersey). Genotypes were imputed from lower-density (8000 markers) SNP chips using FImpute [21], except for 2155 animals, which were directly genotyped with the BovineHD SNP chip (https://www.illumina.com/Documents/products/datasheets/datasheet_bovineHD.pdf (accessed on 2 June 2018)). Quality control for imputation followed Erbe et al. [27]. From the 632,003 SNPs, 578,999 SNPs remained after converting the high-density (HD) genotypes from top–top to forward–forward orientation to match sequence data. The corresponding HD SNP genotypes for the 478 whole-genome sequenced bulls were extracted from their whole-genome sequences based on the BovineHD map positions.

### 2.2. Structural Variation Detection and Genotyping

The process to generate high-quality structural variation (SV) sets is described in Chen et al. [28]. Briefly, Breakdancer [19] and Pindel [20] were first used to generate SVs (including deletions, insertions, inversions and duplications) from every whole-genome sequence (Figure S1). SVs were merged if they overlapped by ≥ 1bp between the two programs. Five SV sets were extracted, further validated and used for imputation from the merged set: (i) SVs detected in the Holstein population (POP_HOL), (ii) SVs detected in the Jersey population (POP_JER), (iii) SVs validated in the 38 twice sequenced individuals (TWICE_SEQ), (iv) SVs validated in the 133 Holstein sire–son pairs (FAM_HOL) and (v) SVs validated in 28 Jersey sire–son pairs (FAM_JER). The validated SV sets can be found in Table S2, and the size distribution of each set can be found in Figure S3.

For each of the 478 animals, SVs from the Pindel output (in VCF format) were compared with each of SV sets above using bedtools [29] to produce a unified set of SV genotypes: if an SV appeared in both the Pindel output and the target SV set, it was treated as a real SV; if an SV appeared only in Pindel output, it was omitted. The output file containing true SVs was then transformed to 012 genotype format using VCFtools [30], where 0 represented the homozygous genotype for the reference allele (no SV), 1 was the heterozygous genotype (heterozygous SV) and 2 was the homozygous genotype for the alternative allele (homozygous SV). In each sample, SV genotypes with a read depth below a threshold of 5 were treated as missing (i.e., coded as 5). Three main SV types, including deletions, inversions and duplications, were tested for imputation accuracy. Insertions were omitted as none were detected in the Jersey individuals.

### 2.3. Structural Variation Imputation Accuracy Assessment

One of the main factors that can influence imputation accuracy is the marker density of the reference population. To investigate the impact on imputation accuracy by reference SNP density, three different SNP panels were used as reference panel scaffolds for imputation and combined with the various SV sets: panel 1, SNP genotypes from Illumina BovineHD BeadChip; panel 2, SNP positions from BovineHD BeadChip with genotypes extracted from a whole-genome sequence; and panel 3, SNP genotypes from a whole-genome sequence whose minor allele frequency (MAF) was greater than 0.05 using VCFtools. The description of these reference panels used for imputation is summarised in Table S3.

The combination of reference panels and SV genotypes from the different SV sets is described in Table S4 and was imputed by two program pipelines: (i) FImpute (version 2.2) [21], a phasing and imputation program, and (ii) the phasing program Eagle (version 2.3) [22] followed by the imputation program Minimac3 [23]. As an SV is a DNA segment rather than a single-point base pair mutation, the definition of the position of the SV may affect its imputation. Therefore, three different SV points (start, middle and end) were used to investigate the impact of position on imputation accuracy in reference panel 1. Using reference panels 2 and 3, SNPs inside SV regions were both included (by default) and excluded for imputation to test which one achieved higher accuracy. To compare the imputation accuracy differences between SNPs and SVs, we also randomly selected 12,000 SNPs from sequencing data and imputed them using panel 2. The flowchart of the pipeline was demonstrated in Figure S2.

A fivefold cross-validation was used to assess the accuracy of imputation. The 478 individuals were randomly separated into five different sets. One set was treated as a testing set, and the other four sets were the reference set. The SV genotypes of individuals in the testing set were masked as missing (coded as 5), whereas all SNP and SV genotypes in the training set were used as the reference group for imputation. This process was repeated five times, testing each set in turn.

Based on the performance of the two programs, we generated a list of SVs imputed with an accuracy above 0.5 from the four different sets with panel 2 using Eagle + Minimac3. After merging all four SV sets by 1 bp overlap, a total of 4361 deletions, 60 inversions and 68 duplications were selected. The merged 578,999 BovineHD SNP and 4489 SV genotypes of the 478 whole-genome sequenced bulls were then used as the reference set to impute SVs on 35,568 dairy cattle. Eagle (version 2.3) [22] was first used to phase haplotypes, and Minimac3 (version 2.0.1) [23] was used for imputation.

### 2.4. Genome-Wide Association Studies

Association analyses were performed for bulls and cows separately using a mixed linear model to investigate whether SVs were significantly associated with dairy traits. SNPs and SVs were tested for significant associations one at a time and for each trait in turn. The following mixed linear model was fitted:

(1) $$y\;=\;1_{n}µ\;+\;Xb+Z_{1}u_{1}+\;Z_{2}u_{2}\;+\;e\;$$

where **y** is a vector of daughter trait deviations (DTD) for a trait; **1_n_** is a vector of ones, *n* is the number of samples and *μ* is the population mean term; **X** contained the genotype indicator variable of candidate markers coded as 0, 1, 2 tested one at a time and the breed indicator variable coded as 0 (pure Jersey), 0.25 (¼ Jersey and ¾ Holstein), 0.5 (half Jersey and half Holstein), 0.75 (¾ Jersey and ¼ Holstein) and 1 (pure Holstein); **b** is the fixed effect vector for the candidate marker and breed; $u_{1}\;$ and $u_{2}\;$ are vectors of random breeding values of individuals and are assumed to be distributed as $u_{1}$ ~ N(0, $G_{1}\sigma_{a1}^{2}$) and $u_{2}$~ N(0,$G_{2}\sigma_{a2}^{2}$), $G_{1}$ being the genomic relationship matrix (GRM) estimated from SNP genotypes and $G_{2}$ being the GRM estimated from SV genotypes; $Z_{1}$ and $Z_{2}$ are incidence matrices for the random effects of SNPs and SVs, respectively; and **e** are residual effects distributed as **e** ~ N(0, $I\sigma_{e}^{2}$). $\sigma_{a1}^{2}$, $\sigma_{a2}^{2}$ and $\sigma_{e}^{2}$ are SNP, SV and residual variances, respectively. The GRM for SNPs and SVs were constructed as described in Yang et al. [31]. 

Meta-analyses for single traits and a multi-trait meta-analysis for production trait were performed following the approach described in Bolormaa et al. [32] based on the marker effects estimated from single-trait GWAS. The multi-trait $\chi^{2}$ statistic was calculated as $\chi^{2}=t_{i}{}^{\prime }V^{-1}t_{i}$, where $t_{i}$ is a 6 × 1 vector of the signed t-values of the ith marker effects for the three traits in bulls and cows, $t_{i}{}^{\prime }$ is the transpose of vector $t_{i}$, and $V^{-1}$ is an inverse of the 6 × 6 correlation matrix, where the correlation is calculated over the 583,488 SNP and SV signed t-values of estimated effects between two traits.

The choice of *p*-value significance level of marker effects was guided by the corresponding Bonferroni correction of the *p*-value of 0.05 as $\frac{0.05}{Number\;of\;tests}$, where the number of tests here is the total number of SNPs and SVs (583,488), so the adjusted *p*-value threshold in this analysis was < 1 × 10^−7^. In the case of no significant SVs identified, less stringent thresholds of *p* < 10^−5^ and *p* < 10^−4^ were applied to allow more SVs to be discovered, although some false positives might arise.

The false discovery rate (FDR) can be used to approximate the number of true positives at the *p*-value thresholds. The FDR for SVs was calculated following the approach proposed by Bolormaa et al. [33]:

(2) $$FDR=\;\frac{P\left(1-\frac{S}{T}\right)}{\left(\frac{S}{T}\right)\left(1-P\right)}\;\;$$

where *p* is the *p*-value threshold, *S* is the number of SVs that were significant at the *p*-value threshold and *T* is the total number of SVs. 

The model was fitted to the data with GCTA v1.26.0 [31]. Manhattan plots and Q–Q plots for GWAS results were created using the R 3.1.0 package *ggplot2*.

### 2.5. Statistical Model for Variance Estimation and Genomic Prediction

The genomic best linear unbiased prediction (GBLUP) model was used to predict GEBVs [34,35] in ASReml4.1 [36]. Two GBLUP models were compared for estimation of variance components and genomic prediction: one model included SNP effects only, and the other included both SNP and SV effects. The latter model is the same as Equation (1), except that there is no fixed effect of candidate markers, whereas the first model considering only the SNP effects further excludes the random effects of SVs ($Z_{2}u_{2}$). 

A 10-fold cross-validation was used to evaluate the genomic prediction accuracy in the bull datasets only. The cross-validation procedure was applied to three sets of bulls: 4057 Holstein bulls, 1058 Jersey bulls and 5115 combined Holstein and Jersey bulls. For each dataset, samples were randomly partitioned into 10 mutually exclusive subsets of approximately equal size for each trait. Each of the 10 subsets was in turn used as validation, while the other 9 subsets were combined and used as training set. Genotypes and phenotypic records were available for the samples in the training set, whereas phenotypes were set to be missing for the samples in the validation set. The cross-validation process was repeated 10 times by re-sampling the sets randomly. The accuracy was calculated as Pearson’s correlation coefficient between the predicted GEBV and the DTDs. The validation was conducted on Holstein, Jersey and mixed Holstein and Jersey breed separately.

## 3. Results

### 3.1. Structural Variant Sets

We generated two population SV sets, POP_HOL and POP_JER, and three validated SV sets, TWICE_SEQ, FAM_HOL and FAM_JER (Table 1). Deletions made up the largest proportion of SV types. Within the population SV sets, there were more SVs detected in Holsteins than in Jerseys likely because the Holstein sample was larger, especially for deletions and inversions. We investigated imputation accuracy in deletions, inversions and duplications from TWICE_SEQ, FAM_HOL, POP_HOL and POP_JER for further imputation analysis. We excluded insertions, of which none were found in the Jersey sets and very few were validated in FAM_HOL. In addition, as only 143 SVs were found in FAM_JER across all types of SVs, this set was also excluded from imputation analysis. The size distribution of each set can be found in Figure S3. 

### 3.2. Imputation Accuracy

**Figure 1:**
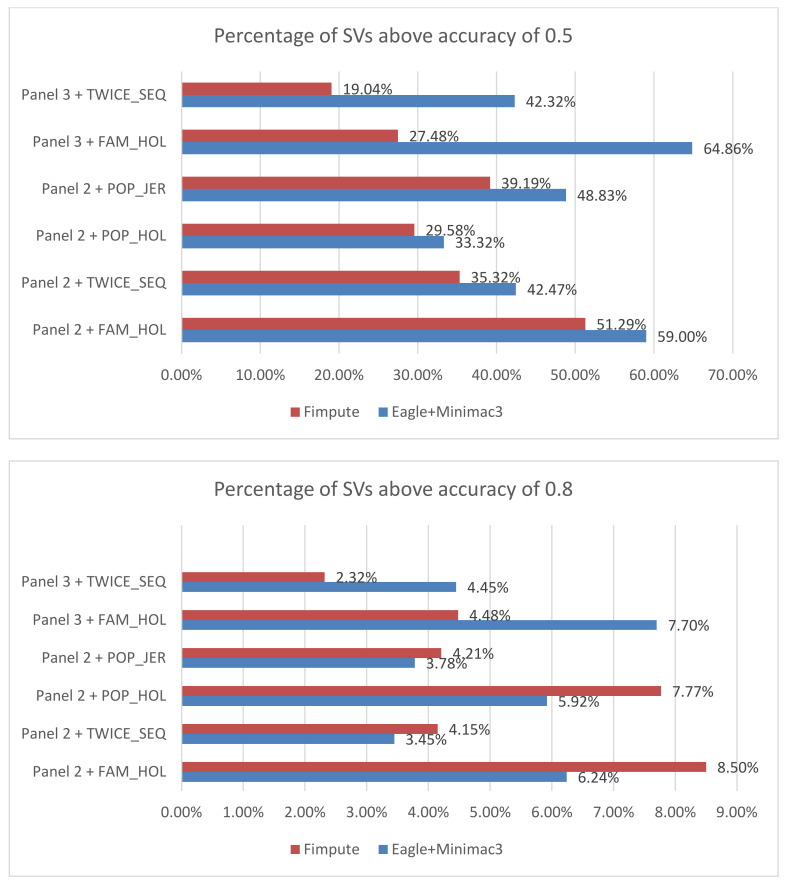
Percentage of deletions with imputation accuracy above thresholds 0.5 (top panel) and 0.8 (bottom panel) for Eagle + Minimac3 and FImpute. *Y* axis: imputation scenarios labelled by reference panel and structural variant (SV) sets.

Overall Eagle + Minimac3 resulted in a larger proportion of SVs with an accuracy above 0.5 than FImpute (Figure 1). Both pipelines reported a similar proportion of SVs with an accuracy above 0.8, while Eagle+Minimac3 performed better with the whole-genome sequence SNP reference (Figure 1). In addition, no large differences by excluding/including SNPs within SVs were found between the two pipelines. Both programs reported the highest accuracy for FAM_HOL and lowest accuracy for POP_HOL. When imputing the other two sets TWICE_SEQ and POP_JER, FImpute reported similar accuracy for the two sets, while Eagle+Minimac3 resulted in lower accuracy in TWICE_SEQ than POP_JER. Imputation results for inversions achieved higher accuracy when using Minimac3 than FImpute.

The distribution of imputation accuracy and imputation accuracy versus MAF for deletions using Eagle + Minimac3 are plotted in Figure S4. For both FAM_HOL and TWICE_SEQ sets, there are more SVs with accuracy >0.5 than the two population sets (Figure S4), suggesting that the validated SV sets contribute to higher-quality SV than other SV sets. In Figure S4, the highest imputation accuracy is found around MAF 0.35 for all the four sets.

### 3.3. GWAS

**Figure 2:**
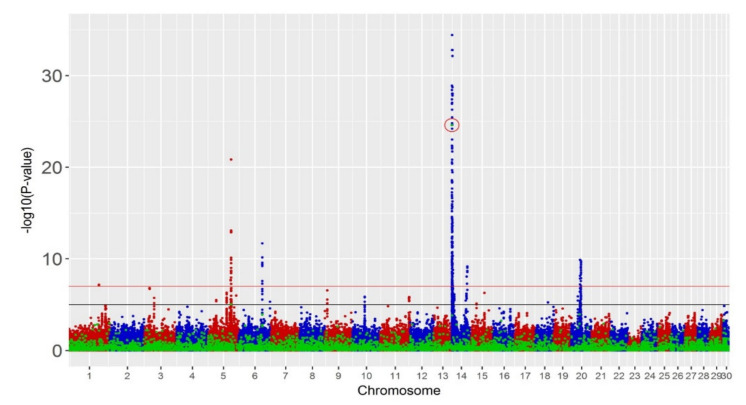
Manhattan plots of meta-analyses of single traits: (top panel) milk yield, (middle) fertility and (bottom) overall type with −log10(*p*-value) on vertical axis and chromosome number on horizontal axis. Red line is the threshold of *p*-value 10^−7^ and black line is the threshold of *p*-value 10^−5^. Blue and red spots represent SNP markers and green spots represent SV markers. Significant SVs with *p*-value < 10^−7^ are circled in red.

We performed single-trait GWAS, which detected a small number of significant SVs (Table 1). One deletion on BTA14 was significant (*p* < 10^−7^) for all the production traits with actual *p*-values 2.2 × 10^−13^, 2.5 × 10^−25^ and 1.6 × 10^−12^ for fat yield (FY), milk yield (MY) and protein yield (PY), respectively. This corresponds to an FDR of 0.04%. With a less stringent threshold (*p* < 10^−5^), one additional deletion on BTA5 was significant for MY (*p* < 9.2 × 10^−6^) with an FDR of 4.49%. No significant SVs were found for fertility and overall type. Hundreds of SNPs were significant for production traits and a smaller set of 34 SNPs for fertility (*p* < 10^−7^). Manhattan plots and Q–Q plots for MY, fertility and overall type are shown in Figure 2 and Figure S5.

**Figure 3:**
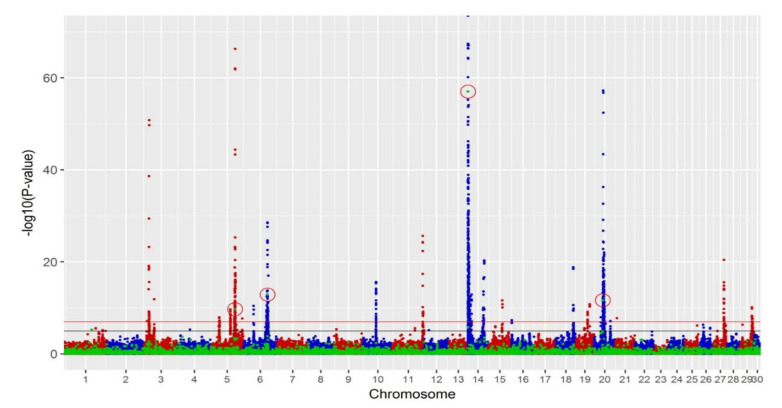
Manhattan plots of production multi-trait meta-analysis with −log_10_(*p*-value) on the vertical axis and chromosome number on the horizontal axis. The red line is the threshold of *p*-value 10^−7^, and the black line is the threshold of *p*-value 10^−5^. Blue and red dots represent SNP markers, and green dots represent SV markers. Significant SVs with *p*-value < 10^−7^ are circled in red. SNPs with *p*-value less than 10^−70^ were not plotted.

A multi-trait meta-analysis for all production traits was performed (Figure 3), and four significant deletions (*p*-value < 10^−7^) were identified with a corresponding FDR of 0.01%, which was lower than for any individual trait. These significant SVs and their overlap with single-trait analyses are summarised in Table 2.

**Figure 4:**
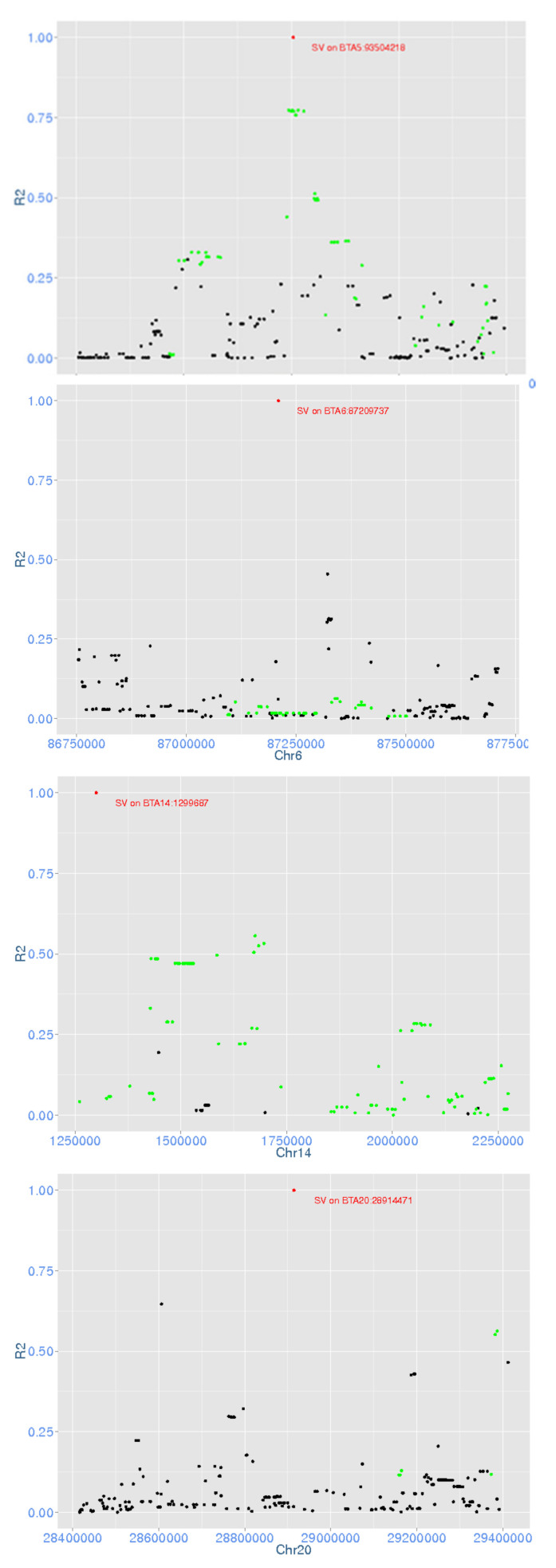
Linkage disequilibrium (LD) of four significant deletions with their surrounding SNPs. In each figure, the red spot is the deletion, black spots are surrounding SNPs and green spots are SNPs that are significant for multi-trait meta-analysis of production traits with *p*-value < 10^−7^.

We investigated whether the four significant deletions were in high linkage disequilibrium (LD) with significant SNP in the same region to determine whether the SV was likely tagging genetic effects not tagged by SNP. The LD between each deletion and the surrounding SNPs (±1 Mb for the one on BTA14 as it is on the start of the chromosome and ±500 kb for the remaining three) were calculated (Figure 4). The two deletions on BTA5 and BTA14 were in high LD (R2 > 0.5) with surrounding SNPs that were also significant in the production multi-trait meta-analysis. On BTA6, although there were many significant SNPs around the deletion, the LD between these SNPs and the deletion were less than 0.1. For the deletion on BTA20, only four SNPs were in high LD, of which three were significant in the production multi-trait meta-analysis.

### 3.4. Variance Components

The genetic and phenotypic variance components for each trait were estimated for Holstein and Jersey bulls separately using models with or without SV effects. Overall, the total genetic variance increased slightly by adding SV information for all traits in both breeds. For all traits except fertility, a larger proportion of genetic variance was explained by SVs in Jersey than in Holsteins. The SVs explained up to 3.8% and 7.3% of the total phenotypic variance for fat and milk yield traits in Holstein and Jersey bulls, respectively (Table 3). While 3.8% and 3.1% of the phenotypic variance was explained by SVs in Holstein bulls for protein yield and fertility, respectively, it was almost zero in Jersey for both traits. No significant SV variance was found for overall type in Holsteins, whereas 2% of the total phenotypic variance was explained in Jerseys but its SE interval included 0 (Table 3).

We also estimated the variance components combining Holstein and Jersey animals, but for bulls and cows separately. For the three production traits, the SVs explained up to 3.6% and 1.3% of the total phenotypic variance for both bulls and cows, respectively. For fertility, SVs explained 3.8% of the total phenotypic variance in bulls, but almost zero for cows, whereas for overall type the SV variance components in both bulls and cows were very close to zero (Table 4). In bulls, although SVs explained similar proportion of the phenotypic variance for fertility (3.8%) and milk yield (3.6%), more genetic variance was captured by SVs for fertility (8.11%) than for milk yield (4.57%).

### 3.5. Genomic Prediction

Three sets of bulls including 4057 Holstein, 1058 Jersey and 5115 combined Holstein and Jersey were tested using models with and without SV effects to compare genomic prediction accuracy. Mean correlations between GEBV and DTDs for the two models in 10 replicates of 10-fold cross-validation are presented in Table 5. For most of the traits, higher prediction accuracies were found in Holstein compared with Jersey bulls except for fat yield. Compared to the model with SNP effects only, there was no consistent increase in accuracy by including SVs in the analysis.

## 4. Discussion

Imputation of SVs from SNP genotype could be a cost-effective way to genotype SVs in a large number of individuals, at least for SVs that are at reasonable MAF in the reference population. We identified 24,908 SVs including deletions, inversions and duplications from four SV sets, which were then imputed using two pipelines. Overall, Eagle2.3-Minimac3 resulted in higher imputation accuracy than FImpute. SVs from FAM_HOL and TWICE_SEQ had a higher proportion of SVs with high imputation accuracy than the other sets. This result suggested that the sire–son validation is useful to remove false positive SVs as concluded in Chen et al. [28]. A high false positive rate for CNVs has been reported [37]. In addition, low-MAF SVs are difficult to impute [18], while common SVs are imputed more accurately and require an SV to be observed in both sire and son, which increases the average MAF for the FAM sets. We found that there were a larger number of rare SV calls in Holstein population calls than in the other sets, which could partly explain why SVs in validated sets achieve higher accuracy (Figure S4).

The comparison between FImpute and Eagle+Minimac3 implied that the latter pipeline can impute SVs more accurately, especially when the SNP markers are very dense (Figure 1). Despite the fact that the percentage of SVs with an accuracy above 0.8 in Eagle+Minimac3 were 1% to 2% lower than FImpute, Eagle+Minimac3 achieved considerably more SVs with an accuracy above 0.5 than FImpute, especially when the imputation was from whole-genome sequence SNPs. It is possible that the imputation accuracy of the SNP themselves could have affected the accuracy of SV imputation. For example, it is known that lower MAF SNPs are less well imputed [24,38]. While the pattern of imputation accuracy versus MAF for SVs was similar to SNP imputation (Figure S4), the overall imputation accuracy for SVs was much lower than for SNPs. This suggests that it is difficult to impute SVs from SNPs based on our current pipeline, yet with the potential to improve SV imputation towards a similar accuracy of SNP. We considered that the way we merged original SV outputs could affect the imputation results. Our threshold of merging SVs with 1 bp overlap could combine discrete mutations with different effects. Our testing with larger overlaps did not substantially change the results.

We performed GWAS in Holstein and Jersey dairy cattle breeds for both bulls and cows to investigate the associations between phenotypes, including production traits, fertility and overall type, and SNP genotypes as well as SV information. In single-trait GWAS, many SNPs that were found significant for FY, MY, PY and fertility were also previously reported in the literature, whereas none was found for overall type. In terms of SV, two deletions were significant for milk yield, whereas in the multi-trait meta-analysis four deletions were significantly associated with at least one production trait. The significant deletions on BTA5 and BTA14 may be mainly due to the high linkage disequilibrium (LD) with their surrounding SNPs. The LD for the two SVs on BTA6 and BTA20 and the surrounding SNPs was not high, even though there were many significant SNPs on these chromosomes. This may indicate that these SV tag genetic information additional to that of SNP.

A list of genes within ±500 kb distance of the four significant SVs (Table S5) was compiled. Several genes have been previously identified for milk production traits. MGST1 on BTA5 has pleiotropic effects on bovine milk composition [39]. A cluster of genes on BTA6 around CSN2 (β-casein) directly regulates milk protein synthesis [40,41]. The histatherin (HSTN) gene was found 5 kb downstream to the SV found on BTA6, which was involved in a genomic rearrangement within the casein gene cluster. As this genomic rearrangement moved HSTN to a regulatory element that is important for CSN2 expression, HSTN is likely to be regulated like the casein genes during the lactation cycle [42]. C14H8orf33 [43], ZNF34 and RPL8 [44,45], which were identified to be associated with milk production traits, are the top three genes that are close to the SV on BTA14. The four SVs found in our study may have potential effects on these genes. The DGAT1 gene located on BTA14:1,795,425–1,804,838 was suggested to be a candidate gene for milk production traits in cattle [46]. The deletion on BTA14: 1,299,687–1,299,831 found in our study was located 500 kb upstream of DGAT1. To investigate whether this deletion was affected by DGAT1, another GWAS model with the fixed effect of this gene added was applied to eliminate the effect of this gene, where the gene was represented as the genotype of the most significant BTA14 SNPs in each individual production trait (BTA14: 1736599). The significant deletion on BTA14 dropped below the significance threshold, suggesting this deletion was likely also tagging DGAT1. 

A small proportion of the total genetic variance was explained by SVs. The proportion explained seemed to also be variable between traits as well as between bulls and cows. The between trait variation can likely be explained by SVs affecting different pathways, whereas the differences between sexes may be due to sampling from unequal population sizes for bulls and cows. Despite some genetic variance being explained by SVs, genomic prediction did not benefit from adding SVs. While the GBLUP model assumes that the SNP effects follow a normal distribution, other models assume that the SNP effects follow a mixture distribution such as BayesR [27] or exponential distributions such as Bayesian Lasso [47]. These models may be more appropriate if SVs have moderate or large effects on the trait. Therefore, we also performed genomic prediction using the BayesR model for 5115 bulls, which yielded very similar accuracy as GBLUP, and no benefit was observed by adding SV information (Table S6). 

In our study, the most likely reason that no significantly higher accuracy was found in the SNP+SV model is that the set of selected SVs were imputed from SNP arrays. Genomic prediction based on imputed whole-genome sequencing data may have very small or no benefit compared to SNP array data [48,49]. SNPs with low MAF are more difficult to impute correctly [50]; yet incorporating rare mutations with low minor allele frequency (MAF) variants can improve the genomic prediction accuracy by up to 30% [49]. As the imputation method used in our study relies on LD information, the selected SVs (accuracy > 0.5) were very likely to be in high LD with the surrounding SNPs. More than half of the originally reported SVs were excluded because SVs with low MAF cannot be imputed well. As many SVs with potential genomic contribution are rare and may have been discarded during the imputation process, we did not observe consistent benefits in genomic prediction by including the imputed SV genotypes. Additionally, the method we used to detect SV with high confidence may lead to high accuracy but low sensitivity, i.e., many potential real SVs were filtered out during the SV detection pipeline [28]. Nevertheless, we find this as preferable to allowing a large number of false positives. The inability to detect SVs in repetitive and poorly mapped regions also limits the detection of the whole-spectrum SV in the bovine genome [51].

Our analyses were based on the UMD3.1 reference assembly and Run5 of the 1000 Bull Genomes Project. More recent runs of the 1000 Bull Genomes Project include a much larger set of Holstein cattle and approximately twice the Jersey samples. This would likely affect imputation accuracy as it would increase the reference dataset. Likewise, the ARS-UCD-1.2 bovine assembly has better resolved repetitive regions of the genome [52]. However, the limited quality of SV detection with paired-end short-read data is expected to remain even with an improved reference and a larger dataset. Third-generation sequencing, including single-molecule real-time (SMRT) sequencing from Pacific BioSciences (PacBio) and Oxford Nanopore sequencing, can produce long reads of several kilobases to facilitate genome assembly. Long reads can help overcome the issue of multiple alignments or misalignments existing with short-reads in repetitive regions because they can span the entire region. Thus, they have been used to improve assembly quality and genome gaps [52,53,54,55] and increase the accuracy and sensitivity of SV detection in low-complexity regions [56]. In addition, several hybrid methods combining reads from next-generation sequencing and single-molecule sequencing have been developed to detect SVs, and many novel SVs within gapped regions and low-complexity regions can now be captured with higher accuracy [54,57,58]. 

## 5. Conclusions

The accurate detection of structural variants from short-read whole-genome sequence and subsequent imputation into animals with only SNP chip genotypes presented a significant challenge. Overall, few SVs were found to be associated significantly with phenotypes, SVs explained a small proportion of the genetic variance, and there was limited influence of SVs on genomic prediction accuracy. Taken together, our findings indicate that the routine application of SVs from short-read sequence data in cattle breeding should not be a priority.

## Figures and Tables

**Table 1:** Number of SNPs and SVs and corresponding FDR below *p*-value thresholds of 10^−7^, 10^−5^ and 10^−4^ for production traits, fertility and overall type for meta-analysis of bull and cow GWAS results.

Trait	*p*-Value Threshold 10^−7^			*p*-Value Threshold 10^−5^			*p*-Value Threshold 10^−4^		
	SNP Number	SV Number	FDR for SV	SNP Number	SV Number	FDR for SV	SNP Number	SV Number	FDR for SV
FY	237	1	0.04%	365	1	4.49%	1159	3	14.95%
MY	289	1	0.04%	505	2	2.24%	1644	2	22.43%
PY	178	1	0.04%	268	1	4.49%	1152	2	14.95%
Fert	34	0		99	0		888	0	
Otype	0	0		11	0		763	2	22.43%

SNP: single-nucleotide polymorphism; SV: structural variation; FDR: false discovery rate; GWAS: genome-wide association study; FY: fat yield; MY: milk yield; PY: protein yield; Fert: fertility; Otype: overall type.

**Table 2:** Significant SVs in the production multi-trait meta-analysis GWAS with *p*-value < 10^−7^.

Chr	Start (bp)	End (bp)	SV Type	*p*-Value	Single-Trait (*p*-Value < 10^−7^)	Single-Trait (*p*-Value < 10^−5^)
Chr5	93,504,218	93,505,234	Deletion	1.9 × 10^−10^		MY
Chr6	87,209,737	87,211,122	Deletion	1.3 × 10^−13^		
Chr14	1,299,687	1,299,831	Deletion	1.0 × 10^−57^	FY, MY, PY	FY, MY, PY
Chr20	28,914,471	28,915,027	Deletion	2.2 × 10^−12^		

**Table 3:** Proportion of SNP and SV variances of total genetic variance and phenotypic variance for milk (MY), fat (FY), protein yield (PY), fertility and overall type (Otype) in Holstein and Jersey bulls.

Breed	Model	Variance Item			Traits		
			FY	MY	PY	Fertility	Otype
Holstein	SNP	$\sigma_{snp}^{2}/\sigma_{p}^{2}$	0.708(0.022)	0.79(0.019)	0.763(0.035)	0.495(0.028)	0.476(0.019)
	SNP + SV	$\sigma_{snp}^{2}/\sigma_{p}^{2}$	0.692(0.027)	0.766(0.023)	0.738(0.041)	0.471(0.033)	0.486(0.024)
		$\sigma_{sv}^{2}/\sigma_{p}^{2}$	0.022(0.021)	0.038(0.019)	0.038(0.03)	0.031(0.024)	0(0.02)
		$\sigma_{sv}^{2}/(\sigma_{snp}^{2}+\sigma_{sv}^{2})$	0.031	0.047	0.049	0.062	0.000
		$(\sigma_{snp}^{2}+\sigma_{sv}^{2})/\sigma_{p}^{2}$	0.715(0.023)	0.804(0.019)	0.777(0.036)	0.502(0.029)	0.486(0.02)
Jersey	SNP	$\sigma_{snp}^{2}/\sigma_{p}^{2}$	0.855(0.037)	0.812(0.043)	0.835(0.08)	0.296(0.065)	0.623(0.038)
	SNP + SV	$\sigma_{snp}^{2}/\sigma_{p}^{2}$	0.841(0.058)	0.759(0.068)	0.842(0.11)	0.32(0.089)	0.609(0.06)
		$\sigma_{sv}^{2}/\sigma_{p}^{2}$	0.019(0.063)	0.073(0.072)	0(0.109)	0(0.094)	0.02(0.068)
		$\sigma_{sv}^{2}/(\sigma_{snp}^{2}+\sigma_{sv}^{2})$	0.022	0.088	0.000	0.000	0.032
		$(\sigma_{snp}^{2}+\sigma_{sv)}^{2}/\sigma_{p}^{2}$	0.86(0.041)	0.833(0.047)	0.842(0.087)	0.32(0.075)	0.629(0.043)

$\sigma_{snp}^{2}$ is the SNP variance, $\sigma_{sv}^{2}$ is the SV variance and $\sigma_{p}^{2}$ is the total phenotypic variance. Standard error is shown in parentheses.

**Table 4:** Proportion of SNP and SV variance components in bulls and cows for fat, milk, protein yield, fertility and overall type traits using model 2.

Bulls	SNP Variance	SV Variance	SNP+SV Variance	SV Variance
Traits	$\sigma_{snp}^{2}/\sigma_{p}^{2}$	$\sigma_{sv}^{2}/\sigma_{p}^{2}$	$(\sigma_{snp}^{2}+\sigma_{sv}^{2})/\sigma_{p}^{2}$	$\sigma_{sv}^{2}/(\sigma_{snp}^{2}+\sigma_{sv}^{2})$
FY	0.728(0.021)	0.014(0.016)	0.743(0.019)	1.94%
Fert	0.433(0.030)	0.038(0.021)	0.471(0.026)	8.11%
MY	0.758(0.02)	0.036(0.016)	0.794(0.017)	4.57%
Otype	0.526(0.036)	0.000(0.026)	0.526(0.033)	0.00%
PY	0.747(0.020)	0.028(0.016)	0.775(0.017)	3.58%
**Cows**				
Traits	$\sigma_{snp}^{2}/\sigma_{p}^{2}$	$\sigma_{sv}^{2}/\sigma_{p}^{2}$	$(\sigma_{snp}^{2}+\sigma_{sv}^{2})/\sigma_{p}^{2}$	$\sigma_{sv}^{2}/(\sigma_{snp}^{2}+\sigma_{sv}^{2})$
FY	0.394(0.009)	0.008(0.004)	0.402(0.009)	1.94%
Fert	0.105(0.008)	0.000(0.004)	0.105(0.007)	0.00%
MY	0.466(0.009)	0.007(0.004)	0.474(0.009)	1.54%
Otype	0.139(0.015)	0.000(0.010)	0.139(0.014)	0.00%
PY	0.366(0.009)	0.013(0.004)	0.379(0.009)	3.53%

The number represents the proportion of variance that each item explained in the phenotypic variance for each trait. SNP: SNP variance; SV: SV variance; SUM: total genetic variance. The last column is the percentage of genetic variance explained by SV.

**Table 5:** Prediction accuracy for each trait in Holstein, Jersey and mixed Holstein and Jersey bulls with SNP only and SNP + SV together in 10-fold cross-validation.

Sample Set	Model	Trait				
		FY	MY	PY	Fertility	OType
Holstein	SNP	0.645(0.008)	0.751(0.007)	0.808(0.005)	0.525(0.011)	0.588(0.014)
	SNP+SV	0.645(0.008)	0.751(0.007)	0.808(0.005)	0.525(0.011)	0.588(0.014)
Jersey	SNP	0.76(0.013)	0.697(0.018)	0.794(0.011)	0.262(0.026)	0.549(0.023)
	SNP+SV	0.76(0.013)	0.698(0.018)	0.795(0.011)	0.262(0.026)	0.55(0.023)
Holstein+Jersey	SNP	0.657(0.013)	0.835(0.005)	0.788(0.007)	0.623(0.009)	0.553(0.013)
	SNP+SV	0.656(0.013)	0.838(0.005)	0.789(0.007)	0.623(0.009)	0.553(0.013)

Standard errors in parentheses.

## Data Availability

Restrictions apply to the availability of these data. Whole-genome sequence data is available to partners of the 1000 Bull Genomes Project, A subset of the sequenced animals are available at the European Variation Archive (PRJEB42783). Phenotype data was obtained from Datagene and may be accessed with their permission (Datagene.com.au (accessed on 19 January 2021)).

## Supplementary Materials

The following are available online at https://www.mdpi.com/2076-2615/11/2/541/s1: Table S1. Summary of genome coverage read depth and insert size of SV for the whole-genome sequenced animals; Table S2. Summary of SV set output; Table S3. Summary of reference panel datasets used for imputation; Table S4. Summary of imputation scenarios with different reference panels and variant sets; Table S5. Genes within ± 500 kb distance of the four significant SVs; Table S6. Genomic prediction accuracies for milk yield, fat yield, protein yield, fertility and overall type of 5115 bulls with BayesR; Figure S1. Flowchart of population SV pipeline; Figure S2. Flowchart of 5-fold cross-validation of imputation pipeline; Figure S3. Plot of size distribution of deletions, insertions, inversions and duplications; Figure S4. Imputation accuracy for deletions in POP_HOL, POP_JER, FAM_HOL and TWICE_SEQ sets; Figure S5. Q-Q plots for meta-analysis of single traits: fat yield, milk yield, protein yield, fertility and overall type.
