# Supplementary material for: Investigating the Effect of Imputed Structural Variants from Whole-Genome Sequence on Genome-Wide Association and Genomic Prediction in Dairy Cattle
Source: Animals (Basel). 2021 Feb 19;11(2):541. doi: 10.3390/ani11020541 (PMC7922624; doi:10.3390/ani11020541)
Supplement: Supplementary file 1 [file animals-11-00541-s001.pdf]

animals

MDPI

# Supplementary Materials: Investigating the Effect of Imputed Structural Variants from Whole-Genome Sequence on Genome-Wide Association and Genomic Prediction in Dairy Cattle

**Long Chen, Jennie E. Pryce, Ben J. Hayes and Hans D. Daetwyler**

**Table S1.** Summary of genome coverage read depth and insert size of SV for the whole-genome sequenced animals.

| Population | Sample Number | Fold Coverage | | | Insert Size (bp) | | |
|---|---|---|---|---|---|---|---|
| | | Min | Mean | Max | Min | Mean | Max |
| Holstein | 415 | 2.21 | 11.46 | 44.52 | 250 | 334.3 | 514 |
| Jersey | 63 | 2.65 | 10.57 | 25.68 | 250 | 352.3 | 502 |

**Table S2.** Summary of SV set output.

| | TWICE_SEQ | FAM_HOL | FAM_JER | POP_HOL | POP_JER |
|---|---|---|---|---|---|
| SV Counts | | | | | |
| Deletions | 10604 | 4182 | 116 | 13119 | 2113 |
| Insertions | 164 | 10 | 0 | 656 | 0 |
| Inversion | 145 | 132 | 15 | 5185 | 571 |
| Duplications | 249 | 347 | 12 | 2074 | 334 |
| SV covered region (Mb) | | | | | |
| Deletions | 4.71 | 3.09 | 0.10 | 9.39 | 2.25 |
| Insertions | 0.01 | 0.00 | 0.00 | 0.04 | 0.00 |
| Inversion | 0.27 | 0.22 | 0.02 | 8.50 | 0.82 |
| Duplications | 0.38 | 0.29 | 0.03 | 2.05 | 0.38 |

TWICE_SEQ: SV set from twice-sequenced individuals. FAM_HOL: SV set from Holstein sire-son pairs. FAM_JER: SV set from Jersey sire-son pairs. POP_HOL: SV set from Holstein population. POP_JER: SV set from Jersey population.

**Table S3.** Summary of reference panel datasets used for imputation.

| Reference Panel * | Number of Markers | Description |
|---|---|---|
| 1. BovineHD (200) | 632,003 | SNPs and genotypes from Illumina BovineHD BeadChip |
| 2. WGS_800K_SNP (478) | 609,259 | BovineHD SNP locations with genotypes from whole-genome sequence |
| 3. WGS_SNP (478) | 12,475,948 | SNPs from whole-genome sequence with MAF >0.05 |

* Panel 1: 200 individuals which have whole-genome sequence and BovineHD BeadChip; Panel 2 and 3: 478 individuals who were whole-genome sequenced only once.

**Table S4.** Summary of imputation scenarios with different reference panels and variant sets.

| | SV Set to be Imputed | | | | SNP |
|---|---|---|---|---|---|
| Reference panel | TWICE_SEQ | FAM_HOL | POP_HOL | POP_JER | Randomly selected SNPs from sequencing data |
| Panel 1 | √ | √ | | | |
| Panel 2 | √ | √ | √ | √ | √ |
| Panel 2 (Exclude) | √ | √ | | | |
| Panel 3 | √ | √ | | | |
| Panel 3 (Exclude) | √ | √ | | | |

Panel 1. SNP genotypes from Illumina BovineHD BeadChip; Panel 2. SNP locations from BovineHD and genotypes from whole-genome sequences; Panel 3. SNP genotypes from whole-genome sequences with minor allele frequency (MAF>0.05). TWICE_SEQ: SV set from twice-sequenced individuals. FAM_HOL: SV set from Holstein sire-son pairs. POP_HOL: SV set from Holstein population. POP_JER: SV set from Jersey population. Exclude represents excluding SNPs inside SV regions from the corresponding panel. The randomly selected SNPs were retrieved from Panel 3 WGS SNPs (MAF>0.05).

**Table S5.** Genes within ±500 kb distance of the four significant SVs.

| Gene | Chr | Start | End | SV Position | | | Distance in KB |
|---|---|---|---|---|---|---|---|
| LMO3 | Chr5 | 93693960 | 93757645 | Chr5 | 93504218 | 93505234 | 188.726 |
| MGST1 | Chr5 | 93926898 | 93942185 | | | | 421.664 |
| LOC781988 | Chr6 | 86676446 | 86713666 | Chr6 | 87209737 | 87211122 | -496.071 |
| UGT2A3 | Chr6 | 86810491 | 86835733 | | | | -374.004 |
| SULT1B1 | Chr6 | 86917971 | 86942950 | | | | -266.787 |
| SULT1E1 | Chr6 | 87035918 | 87094959 | | | | -114.778 |
| CSN1S1 | Chr6 | 87141555 | 87159096 | | | | -50.641 |
| CSN2 | Chr6 | 87179506 | 87186022 | | | | -23.715 |
| HSTN | Chr6 | 87189735 | 87204440 | | | | -5.297 |
| STATH | Chr6 | 87229655 | 87239003 | | | | 18.533 |
| CSN1S2 | Chr6 | 87262456 | 87280936 | | | | 51.334 |
| ODAM | Chr6 | 87327027 | 87335880 | | | | 115.905 |
| CSN3 | Chr6 | 87378400 | 87392750 | | | | 167.278 |
| CABS1 | Chr6 | 87469515 | 87471615 | | | | 258.393 |
| AMBN | Chr6 | 87694424 | 87705731 | | | | 483.302 |
| C14H8orf33 | Chr14 | 1487365 | 1489409 | Chr14 | 1299687 | 1299831 | 187.534 |
| ZNF34 | Chr14 | 1491980 | 1497904 | | | | 192.149 |
| RPL8 | Chr14 | 1505029 | 1507626 | | | | 205.198 |
| COMMD5 | Chr14 | 1531490 | 1533526 | | | | 231.659 |
| ARHGAP39 | Chr14 | 1563865 | 1600378 | | | | 264.034 |
| MIR2308 | Chr14 | 1566932 | 1567001 | | | | 267.101 |
| C14H8orf82 | Chr14 | 1602473 | 1604999 | | | | 302.642 |
| LRRC24 | Chr14 | 1604104 | 1609477 | | | | 304.273 |
| LRRC14 | Chr14 | 1610261 | 1613725 | | | | 310.43 |
| RECQL4 | Chr14 | 1614026 | 1620509 | | | | 314.195 |
| MFSD3 | Chr14 | 1620565 | 1622643 | | | | 320.734 |
| GPT | Chr14 | 1623901 | 1626907 | | | | 324.07 |
| PPP1R16A | Chr14 | 1628813 | 1633988 | | | | 328.982 |
| FOXH1 | Chr14 | 1654700 | 1656256 | | | | 354.869 |
| KIFC2 | Chr14 | 1656360 | 1663803 | | | | 356.529 |
| CYHR1 | Chr14 | 1664422 | 1665518 | | | | 364.591 |

| | | | | | | | |
|---|---|---|---|---|---|---|---|
| CYHR1 | Chr14 | 1674620 | 1677519 | | | | 374.789 |
| TONSL | Chr14 | 1681493 | 1692499 | | | | 381.662 |
| VPS28 | Chr14 | 1693642 | 1698621 | | | | 393.811 |
| SLC39A4 | Chr14 | 1719731 | 1724221 | | | | 419.9 |
| CPSF1 | Chr14 | 1728206 | 1742668 | | | | 428.375 |
| ADCK5 | Chr14 | 1742710 | 1756301 | | | | 442.879 |
| SLC52A2 | Chr14 | 1763992 | 1766621 | | | | 464.161 |
| SCRT1 | Chr14 | 1782900 | 1790463 | | | | 483.069 |
| DGAT1 | Chr14 | 1795424 | 1804838 | | | | 495.593 |
| PARP8 | Chr20 | 28315520 | 28506629 | Chr20 | 28914471 | 28915027 | -407.842 |
| HCN1 | Chr20 | 29121454 | 29567228 | | | | 206.427 |

**Table S6.** Genomic prediction accuracies (standard errors) from cross-validations for milk yield, fat yield, protein yield, fertility and overall type of 5115 bulls with BayesR.

| **Sample Set** | **Model** | **Trait** | | | | |
|---|---|---|---|---|---|---|
| | | FY | MY | PY | Fertility | Overall type |
| Holstein + Jersey | SNP | 0.773(0.013) | 0.929(0.001) | 0.902(0.003) | 0.618(0.034) | 0.561(0.030) |
| | SNP+SV | 0.773(0.013) | 0.929(0.001) | 0.902(0.003) | 0.618(0.034) | 0.561(0.030) |

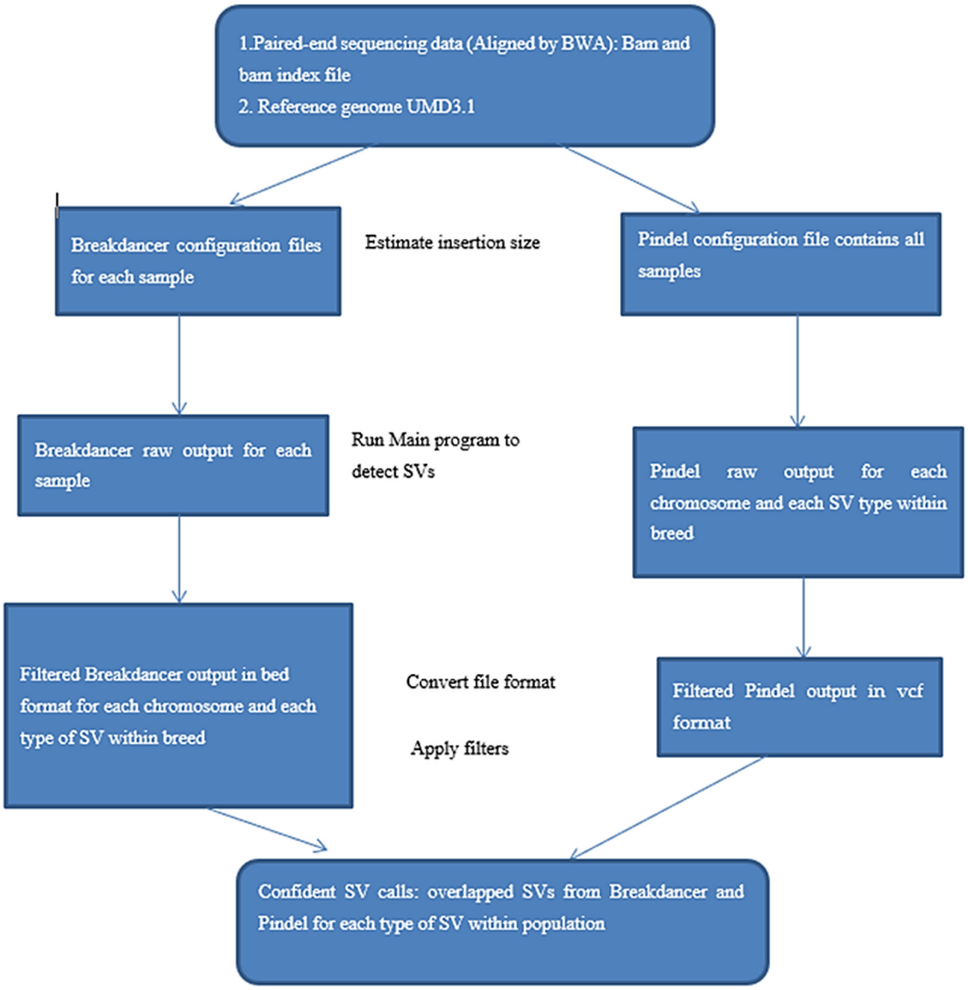


**Figure S1.** Flowchart for population SV pipeline.This flowchart illustrates the pipeline for generating population SV calls for Holstein and Jersey populations. In the filtering step, for both Breakdancer and Pindel, only SVs that were detected in at least 2 animals and > 50 bp regions from genome gaps were included. A few filters were further applied to the raw output of Pindel by setting: minimum coverage as 1; minimum SV event size as 50; minimum supporting reads as 5; maximum supporting reads as twice as the genome coverage for deletions, inversions and insertions and as 4 times of the genome coverage for tandem duplications. The final confident SV sets were then overlapped from filtered Breakdancer and Pindel output set by each SV type, respectively.

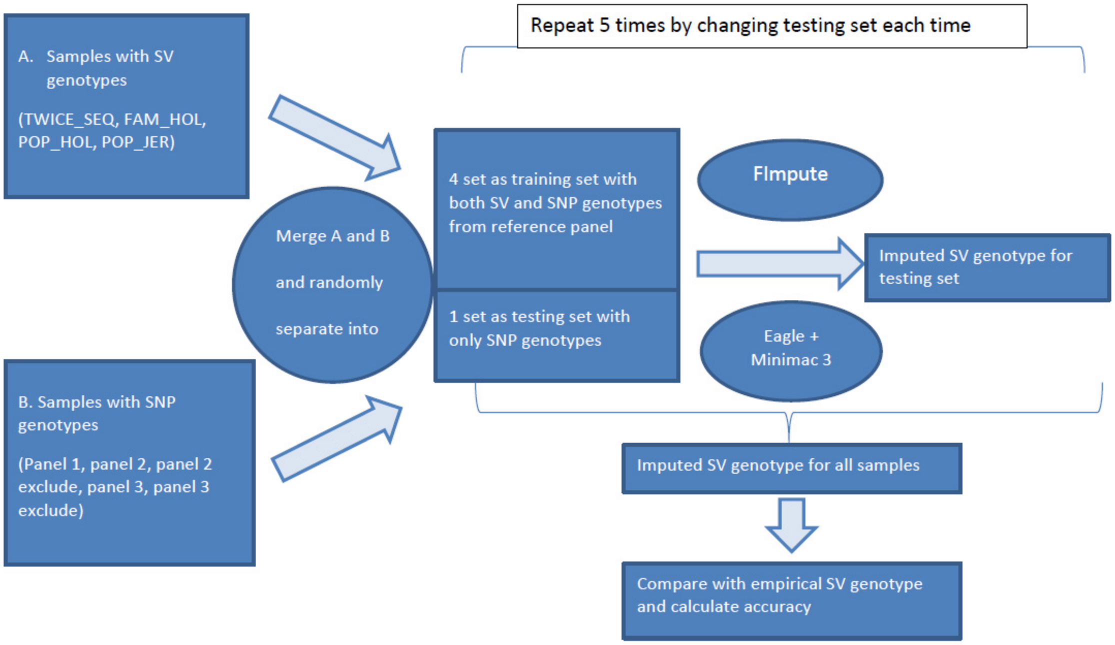


**Figure S2.** Flowchart of 5-fold cross-validation of imputation pipeline.

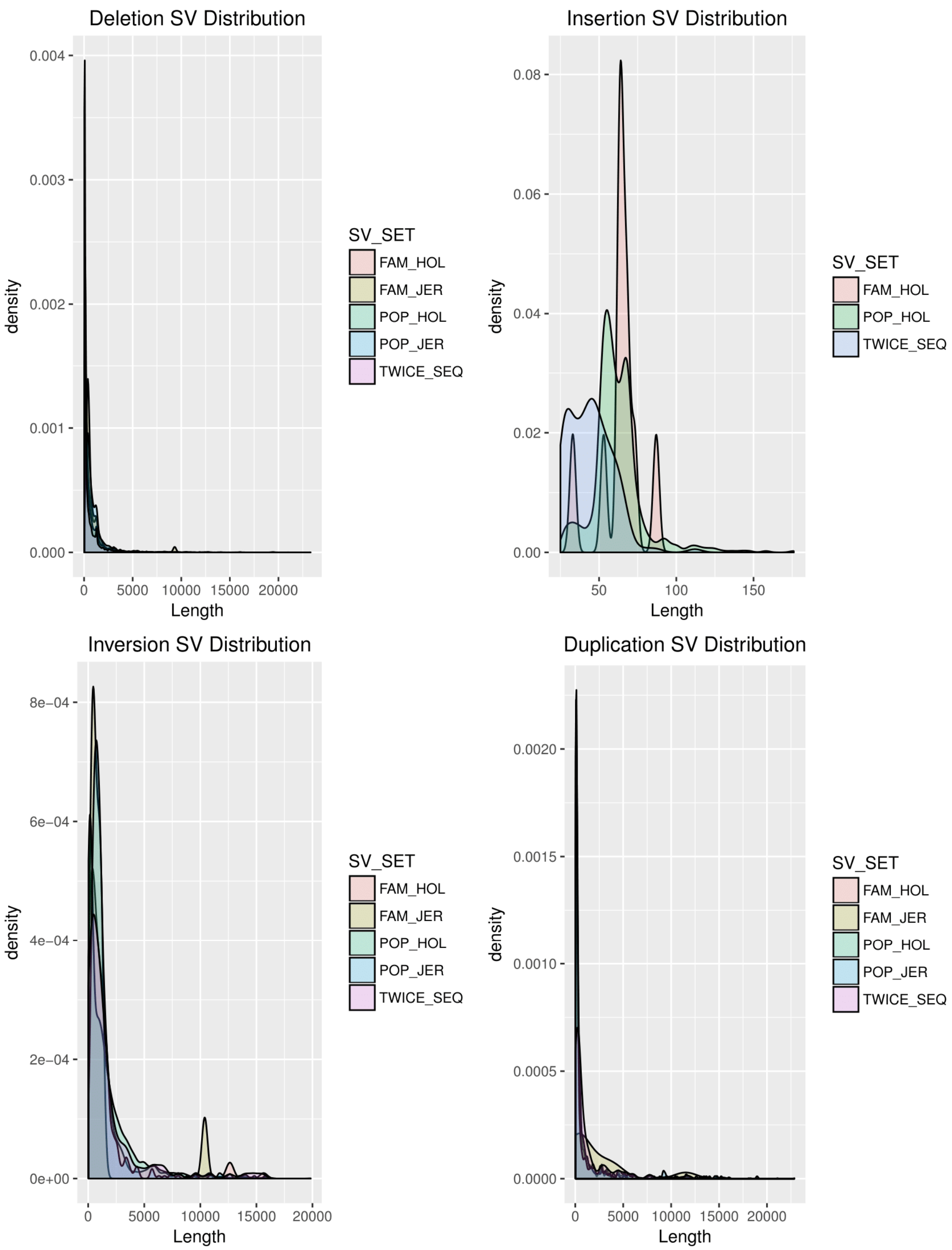


**Figure S3.** Plot of size distribution in base pairs of deletions, insertions, inversions and duplications in FAM_HOL, FAM_JER, POP_HOL, POP_JER and TWICE_SEQ.

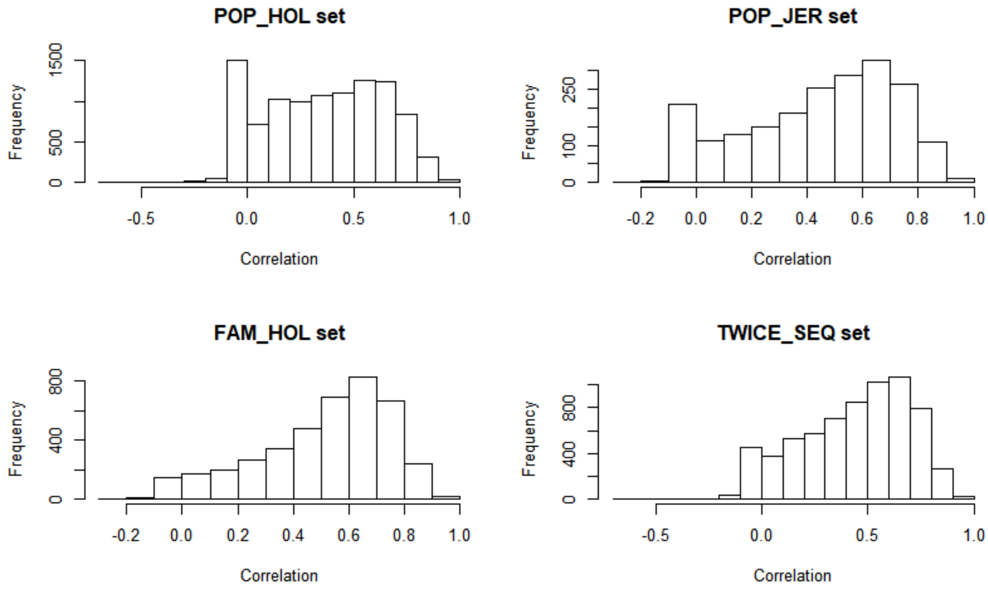


b.

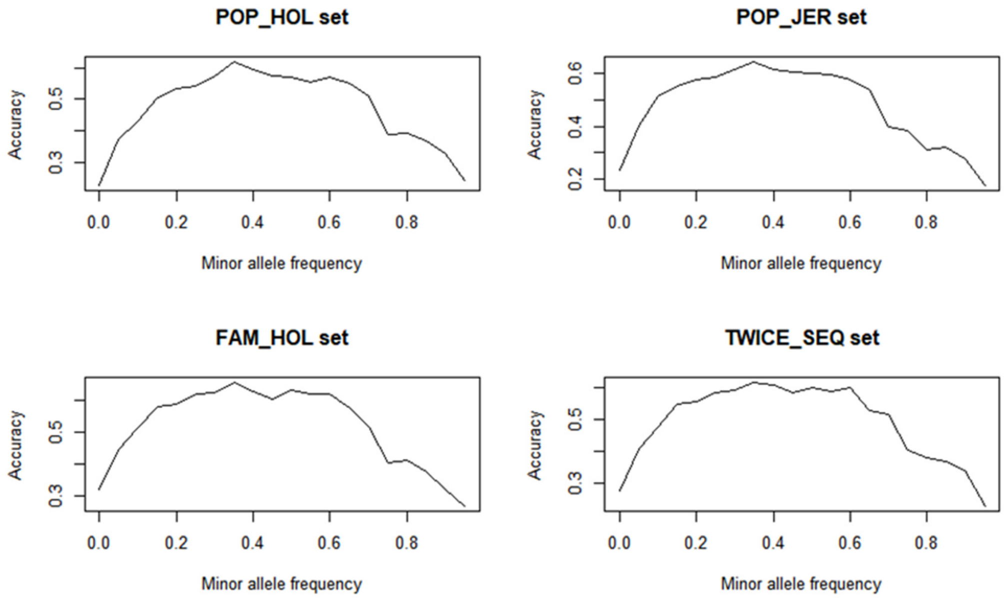


**Figure S4.** Imputation accuracy for deletions in POP_HOL, POP_JER, FAM_HOL and TWICE_SEQ sets. a. Distribution of imputation accuracy. b. Imputation accuracy versus MAF where imputation accuracy is calculated as the mean in each 0.05 MAF bin.

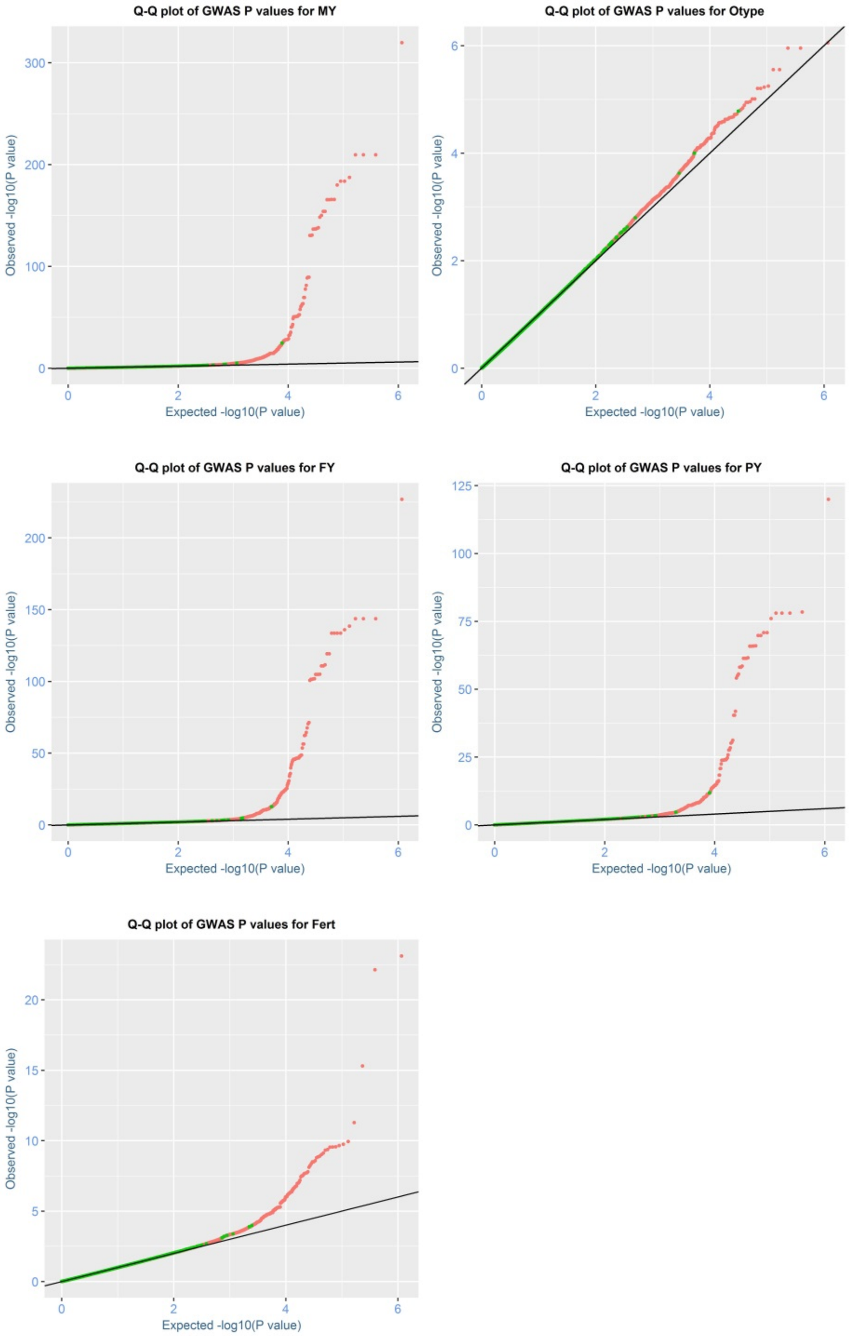


**Figure S5.** QQ plots for meta-analysis of single traits: fat yield, milk yield, protein yield, fertility and overall type with observed -log10(P-value) on vertical axis and expected -log10(P-value) on horizontal axis. Red dots are SNPs and green dots are SVs.
